# The LEDA Score: An Integrated Echocardiographic Score for the Diagnosis of HFpEF

**DOI:** 10.1016/j.jacadv.2026.103246

**Published:** 2026-09-18

**Authors:** Przemysław Palka, Roland Hilling-Smith, Brendan Bell, Alex Incani, Karl Poon, Rohan Swann, Sean Allwood, Alexander Moore, Chris Bian, Aleksandra Lange

**Affiliations:** a St Andrew’s War Memorial Hospital, Spring Hill, Queensland, Australia; b Mater Private Hospital, South Brisbane, Queensland, Australia; c The University of Queensland, St Lucia, Queensland, Australia; d Queensland University of Technology, Brisbane, Queensland, Australia

**Keywords:** diagnostic accuracy, HFpEF, invasive hemodynamics, LEDA score, left-sided filling pressure, ventricular-atrial coupling

## Abstract

**Background:**

Diagnosis of heart failure with preserved ejection fraction (HFpEF) remains challenging because clinical findings may not reflect hemodynamic disease expression.

**Objectives:**

The aim of the study was to evaluate the diagnostic performance of LEDA (Left ventricular concentricity, E/e′, left ventricle-to-left atrium Diastasis volume ratio, left Atrial reservoir function), an integrated echocardiographic score, for identifying HFpEF and elevated left-sided filling pressure (LSFP).

**Methods:**

In this prospective single-center study, 171 selected patients with unexplained dyspnea underwent echocardiography and an invasive hemodynamic assessment within 24 hours. HFpEF was defined by symptoms or signs of heart failure with elevated levels of N-terminal pro–B-type natriuretic peptide and/or invasively measured LSFP ≥15 mm Hg. LEDA integrates left ventricular concentricity, E/e′, left ventricular-left atrial diastasis coupling, and left atrial reservoir function. Diagnostic performance was compared with H_2_FPEF (Heavy, Hypertensive, Atrial fibrillation, Pulmonary hypertension, Elder, Filling pressure score) and HFA-PEFF (Heart Failure Association Pre-test assessment, Echocardiography & natriuretic peptide, Functional testing, Final etiology diagnostic algorithm); 2025 American Society of Echocardiography diastolic criteria were analyzed as an echocardiographic filling-pressure framework rather than as a standalone HFpEF diagnostic score.

**Results:**

HFpEF was present in 42% of patients. LEDA showed higher discrimination for HFpEF than for H_2_FPEF, HFA-PEFF, and American Society of Echocardiography criteria, with area under the curve of 0.85, 0.74, 0.74, and 0.61, respectively (*P* ≤ 0.010). At LEDA ≥3, the positive likelihood ratio was 7.4, compared with 3.0, 1.8, and 1.6, respectively. For invasive LSFP endpoints, LEDA achieved area under the curve up to 0.90, specificity up to 95.5%, and positive likelihood ratio up to 16.3.

**Conclusions:**

In this selected cohort undergoing an invasive hemodynamic assessment for diagnostic uncertainty, LEDA showed stronger diagnostic performance than contemporary HFpEF diagnostic algorithms and an echocardiographic filling-pressure framework. External validation is required before application to broader outpatient populations.

Heart failure with preserved ejection fraction (HFpEF) is a heterogeneous syndrome in which symptoms, comorbidity burden, natriuretic peptides, and resting echocardiographic findings may not consistently reflect hemodynamic disease status.^1^^,^^2^ Elevated left-sided filling pressure (LSFP) remains central to diagnosis, and invasive hemodynamic assessment remains important in patients with unexplained dyspnea and diagnostic uncertainty.^3^

Current approaches include clinical-echocardiographic algorithms such as H_2_FPEF (Heavy, Hypertensive, Atrial fibrillation, Pulmonary hypertension, Elder, Filling pressure score) and HFA-PEFF (Heart Failure Association Pre-test assessment, Echocardiography & natriuretic peptide, Functional testing, Final etiology diagnostic algorithm) and echocardiographic criteria for estimating LSFP.^4^, ^5^, ^6^ However, these tools may leave patients in intermediate categories, disagree with each other, or perform less consistently in selected phenotypes, including atrial fibrillation.^7^, ^8^, ^9^, ^10^, ^11^ Echocardiographic filling-pressure algorithms are useful but should not be used in isolation to exclude hemodynamically confirmed HFpEF.^8^

Left ventricle (LV)-left atrium (LA) interaction, LA remodeling, and ventricular-atrial coupling are increasingly recognized as determinants of LSFP.^12^, ^13^, ^14^ We therefore developed LEDA (Left ventricular concentricity, E/e′, left ventricle-to-left atrium Diastasis volume ratio, left Atrial reservoir function), an integrated echocardiographic score combining LV concentricity, ratio of early mitral inflow velocity to mitral annular early diastolic velocity (E/e′), LV-to-LA diastasis volume ratio, and LA reservoir function. In selected patients with unexplained dyspnea and paired invasive assessment, we evaluated LEDA for identifying clinical HFpEF and invasive LSFP elevation.

## Methods

### Study population

This prospective single-center study included consecutive patients referred for an invasive hemodynamic assessment of unexplained dyspnea after persistent diagnostic uncertainty despite clinical and noninvasive evaluation. Between February 2021 and April 2026, 241 patients were screened; 171 with a preserved LV ejection fraction and paired transthoracic echocardiography and an invasive hemodynamic assessment within 24 hours formed the final analytic cohort (Supplemental Figure 1).

### Cardiac catheterization

Hemodynamic analyses were performed separately from the clinical HFpEF diagnosis using invasively measured LSFP thresholds. In the absence of significant mitral valve or pulmonary vascular disease, pulmonary capillary wedge pressure and direct LA pressure were considered hemodynamically equivalent surrogates of mean LA pressure and LV filling pressure.^15^, ^16^, ^17^, ^18^ Hemodynamically unstable patients, including those requiring inotropic support, mechanical circulatory support, or mechanical ventilation, and those with acute coronary syndrome were excluded. Invasive assessment was also deferred in the presence of relative contraindications, as detailed in the Supplemental Appendix. In selected complex cases (n = 9, 5.3%), direct LA pressure was measured retrogradely.^19^ To minimize incorporation bias, diagnostic performance was also assessed using invasive LSFP endpoints alone, defined independently of echocardiographic variables and without reference to LEDA components.

### Ethics

The study complied with the Declaration of Helsinki. The protocol was approved by the UnitingCare Human Research Ethics Committee (2021.01.339 and 2024.13.407), and all patients provided written informed consent.

### Definition of HFpEF and hemodynamic endpoints

HFpEF was defined by signs and/or symptoms of heart failure with elevated levels of N-terminal pro–B-type natriuretic peptide (NT-proBNP) and/or invasively measured LSFP ≥15 mm Hg, consistent with contemporary guideline frameworks.^17^^,^^18^^,^^20^^,^^21^ NT-proBNP thresholds were adjusted for age and rhythm status, including higher thresholds in atrial fibrillation.^22^^,^^23^

Invasive hemodynamic measurements were the reference standard.^24^^,^^25^ Two hemodynamic endpoints were analyzed: LSFP >12 mm Hg and LSFP ≥15 mm Hg. For these analyses, patients were classified across the entire cohort according to measured LSFP thresholds, independent of clinical HFpEF group assignment; thus, LSFP ≥15 mm Hg performance reflects discrimination of invasive LSFP ≥15 vs <15 mm Hg, not discrimination between clinical HFpEF and non-HFpEF groups.

### Echocardiographic assessment

Comprehensive transthoracic echocardiographic studies were acquired using Philips EPIQ CVx, Philips Affiniti CVx, and Siemens SC2000 systems according to guideline recommendations.^26^, ^27^, ^28^, ^29^ LV and LA strain analyses were performed using the integrated Philips AutoStrain application, powered by TOMTEC technology, when suitable Philips-acquired images were available. Offline LV and LA volume measurements were performed using syngoDynamics software. Image quality was assessed qualitatively by the acquiring sonographer and/or reporting cardiologist. Poor image quality indicated technically limited imaging with sufficient quality to measure required variables; nondiagnostic imaging indicated inability to measure key study variables, particularly LV and LA volumes. All echocardiographic measurements were performed by experienced readers blinded to invasive hemodynamic results.

LEDA (0-4) comprised 4 components: LV end-diastolic volume index divided by mean wall thickness (L), E/e′ (E), LV-to-LA diastasis volume ratio (D), and LA reservoir function calculated as: (LA maximum volume − LA minimum volume) / LA maximum volume (A).^30^ Abnormal thresholds were L <50, E >9, D <1.25, and A <0.40. These thresholds were selected for stage C symptomatic HFpEF with hemodynamic LSFP elevation,^14^ whereas earlier thresholds from hypertensive heart disease reflected stage B remodeling.^31^ Normal values for LEDA scoring were L ≥50, E/e′ ≤9, D ≥1.25, and A ≥0.40. Each abnormal component contributed 1 point.

LEDA was compared with established HFpEF diagnostic algorithms, H_2_FPEF^4^ and HFA-PEFF.^5^ The 2025 American Society of Echocardiography (ASE) diastolic criteria were analyzed as an echocardiographic framework for estimating LA pressure/LSFP, not as a standalone HFpEF diagnostic score.^6^

### Statistical analysis

Continuous variables are presented as mean ± SD or median (IQR), as appropriate. Categorical variables are expressed as counts and percentages. Between-group comparisons were performed using Student’s *t*-test or the Mann-Whitney U test for continuous variables, and the chi-square test or Fisher exact test for categorical variables. For box-and-whisker plots, boxes represent the median and interquartile range, whiskers indicate the most extreme nonoutlier values according to the Tukey method, and overlaid circles represent individual observations.

Associations between continuous variables were assessed using Pearson correlation coefficients for normally distributed data and Spearman rank correlation coefficients for non-normally distributed or ordinal data. Diagnostic accuracy was evaluated using receiver operating characteristic curve analysis, with calculation of the area under the curve (AUC) and corresponding 95% CIs. AUC confidence intervals were estimated using bootstrap resampling. Comparisons between AUCs were performed using the DeLong method. Sensitivity, specificity, positive and negative likelihood ratios (LRs), and diagnostic accuracy were calculated at predefined thresholds. Diagnostic performance was assessed for clinical HFpEF and for invasive hemodynamic endpoints, including LSFP >12 mm Hg and LSFP ≥15 mm Hg. Multivariable logistic regression was used to evaluate whether LEDA was independently associated with HFpEF after adjustment for clinically relevant covariates, including age, sex, atrial fibrillation, hypertension, diabetes mellitus type 2, coronary artery disease, body mass index, and estimated glomerular filtration rate. Results are presented as ORs with 95% CIs. These analyses were inferential and were not intended to develop or validate a clinical prediction model. Additional sensitivity analyses were performed to assess whether the association between LEDA and HFpEF or invasive LSFP was independent of atrial fibrillation status. Atrial fibrillation was analyzed using 2 definitions: any history of atrial fibrillation, including paroxysmal, persistent, or permanent/chronic atrial fibrillation; and persistent/current atrial fibrillation present at the time of hemodynamic and echocardiographic assessment. Logistic regression models were used for HFpEF diagnosis and elevated LSFP ≥15 mm Hg, and linear regression models were used for continuous invasive LSFP. Interaction terms between LEDA and atrial fibrillation status were tested to assess whether rhythm status modified the association between LEDA and diagnostic or hemodynamic outcomes. Bootstrap resampling with 1,000 iterations was used to estimate confidence intervals for diagnostic and reclassification measures and to assess the stability of the findings. Because the study was not designed to develop a formal prediction model, optimism-corrected model performance estimates were not the primary focus. Reclassification analyses were considered exploratory. Because overall net reclassification measures may be difficult to interpret in modest-sized cohorts, reclassification results were interpreted descriptively, and primary emphasis was placed on diagnostic discrimination, LRs, and decision curve analysis. Clinical utility was assessed using decision curve analysis, estimating net benefit across threshold probabilities and comparing LEDA with the clinical model. Additional exploratory analyses were performed to characterize the distribution of LEDA scores, the frequency and pairwise overlap of the 4 LEDA components, and the ability of LEDA categories to reassign patients in intermediate categories of H_2_FPEF (scores 2-5), HFA-PEFF (scores 2-4), and ASE 2025 LA pressure grading (grade 1). Pairwise component overlap was assessed using 2×2 tables and phi coefficients, and concordance with the final HFpEF status was assessed descriptively. The first 53 consecutive patients were used as a derivation cohort. The subsequent 118 patients were analyzed prospectively as a validation cohort using predefined LEDA components and thresholds without modification. Reproducibility of LV-LA diastasis volume ratio was assessed in 27 patients using intraclass correlation coefficients, Bland-Altman bias and 95% limits of agreement, coefficients of variation, and agreement for D <1.25. No formal a priori sample-size calculation was performed for the full LEDA score; the original LV-LA diastasis component was supported by a provisional power calculation in prior work.^14^

Analyses were performed using JMP 18/JMP Pro 19.1.1 and MedCalc 22.009; 2-sided *P* < 0.05 was significant.

## Results

### Study population

The final analytic cohort included 171 patients; 72 (42%) met the criteria for HFpEF. Baseline characteristics are summarized in Table 1. Patients with HFpEF had higher body mass index (30.0 ± 5.8 vs 28.2 ± 5.5), more atrial fibrillation (61.1% vs 20.2%), diabetes type 2 (25.0% vs 11.1%), obstructive sleep apnea (38.9% vs 19.2%), chronic kidney disease stage 3 (55.6% vs 26.3%), higher NT-proBNP (533 [227-1,334] vs 195 [101-382] pg/mL), and more NYHA functional class III-IV symptoms (65.3% vs 21.2%; all *P* ≤ 0.017). Echocardiographic comparisons are provided in Supplemental Table 1.

**Table 1:** Baseline Characteristics of the Study Population

	No HFpEF (n = 99)	HFpEF (n = 72)	*P* Value
Demographics			
Age, y	73.6 ± 9.2	74.9 ± 8.4	0.340
Female, n (%)	58 (58.6)	41 (56.9)	0.830
Body mass index, kg/m^2^	28.2 ± 5.5	30.0 ± 5.8	0.037
Cardiac rhythm and comorbidities			
Any atrial fibrillation, n (%)	20 (20.2)	44 (61.1)	<0.001
Nonparoxysmal atrial fibrillation, n (%)	7 (7.1)	18 (25.0)	0.001
Coronary artery disease, n (%)^a^	31 (31.3)	25 (34.7)	0.640
Diabetes mellitus type 2, n (%)	11 (11.1)	18 (25.0)	0.017
Hypertension, n (%)	69 (69.7)	57 (79.2)	0.170
Smoking history, n (%)	15 (15.2)	15 (20.8)	0.340
Obstructive sleep apnea, n (%)	19 (19.2)	28 (38.9)	0.004
Stroke history, n (%)	3 (3.0)	5 (6.9)	0.280
Cancer history, n (%)	22 (22.2)	22 (30.6)	0.220
Clinical status			
NYHA functional class	2.2 ± 0.5	2.7 ± 0.6	<0.001
NYHA functional class III-IV, n (%)	21 (21.2)	47 (65.3)	<0.001
Systolic blood pressure, mm Hg	128.0 ± 22.4	133.9 ± 24.7	0.110
Diastolic blood pressure, mm Hg	66.1 ± 10.1	68.0 ± 12.5	0.300
Heart rate, beats/min	65.0 ± 10.5	69.4 ± 10.4	0.007
Electrocardiogram (QRS duration), ms	98.9 ± 24.1	106.0 ± 24.9	0.065
Laboratory parameters			
Estimated glomerular filtration rate, mL/min/1.73 m^2^	69.9 ± 16.8	59.8 ± 20.0	<0.001
Chronic kidney disease stage ≥3, n (%)	26 (26.3)	40 (55.6)	<0.001
Hemoglobin, g/L	131.1 ± 18.7	126.0 ± 17.2	0.071
Iron deficiency, n (%)	45 (46.9)	42 (63.6)	0.036
NT-proBNP, pg/mL	195 [101-382]	533 [227-1,334]	<0.001
Hemodynamics			
LSFP, mm Hg	9.4 ± 2.8	18.0 ± 4.8	<0.001
LSFP >12 mm Hg, n (%)	14 (14.1)	68 (94.4)	<0.001
LSFP ≥15 mm Hg, n (%)	0 (0.0)	60 (83.3)	<0.001

Values are mean ± SD, median [IQR], or n (%).

HFpEF = heart failure with preserved ejection fraction; LSFP = left-sided filling pressure; NT proBNP = N-terminal pro–B-type natriuretic peptide.

a Coronary artery disease was defined as a history of type 1 myocardial infarction, percutaneous coronary intervention, and/or coronary artery bypass surgery.

### Association with hemodynamic congestion

Invasive LSFP increased across LEDA score categories (Figure 1), with patients with HFpEF clustering at higher LEDA scores and higher filling pressures. This ordinal association was also supported by a correlation analysis between LEDA score and invasive LSFP (Pearson r = 0.64; 95% CI: 0.54-0.72; *P* < 0.001; Spearman ρ = 0.67).

**Figure 1:**
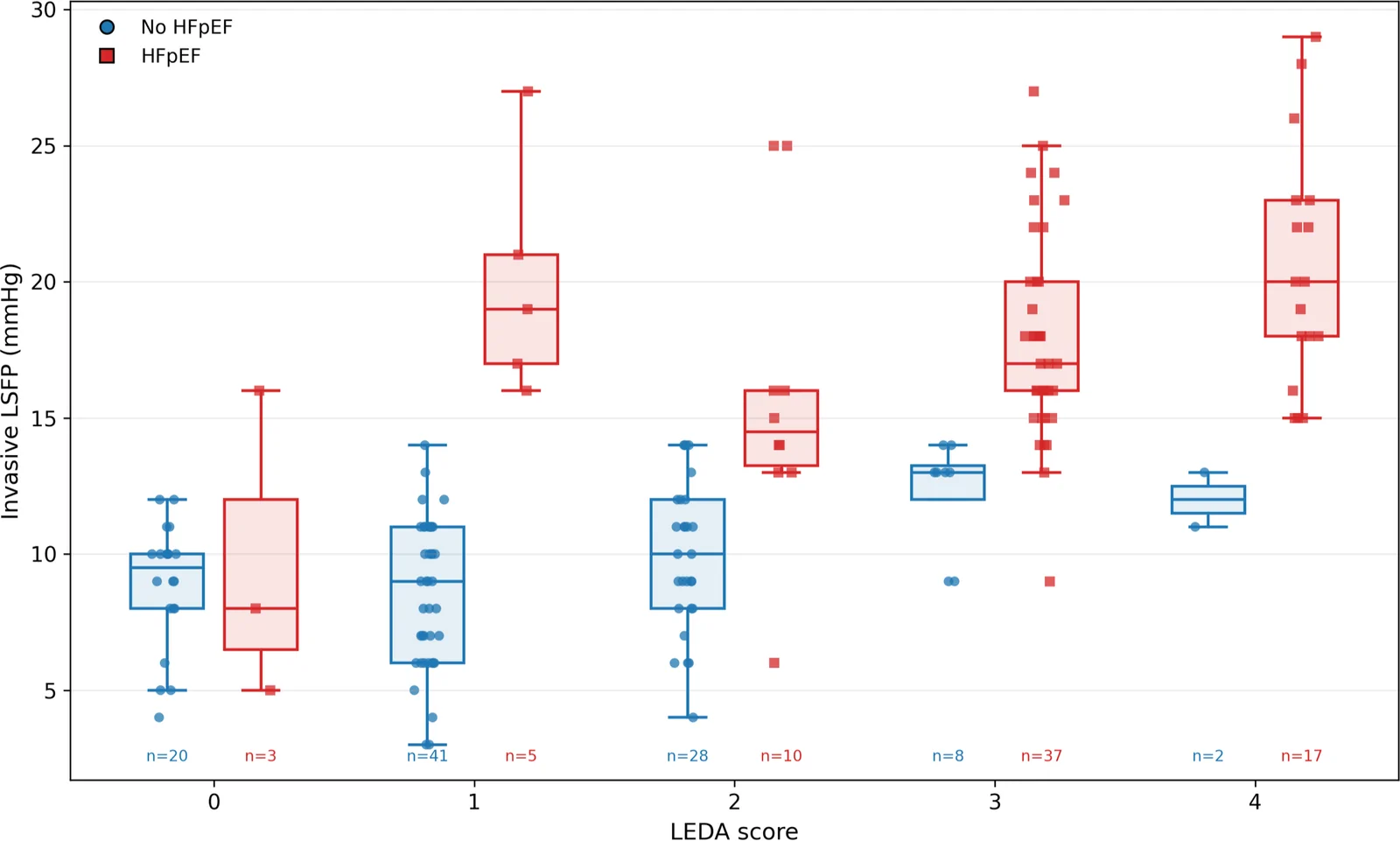
LSFP by LEDA Score and HFpEF Status Box-and-whisker plots showing invasively measured LSFP across LEDA scores from 0 to 4, stratified by HFpEF status. Higher LEDA scores were associated with higher LSFP, with patients with HFpEF clustering at higher LEDA scores and higher filling pressures. Boxes represent median and interquartile range; whiskers indicate the most extreme nonoutlier values according to the Tukey method; blue circles represent patients without HFpEF, and red squares represent patients with HFpEF. HFpEF = heart failure with preserved ejection fraction; LEDA = Left ventricular concentricity, E/e′, left ventricle-to-left atrium Diastasis volume ratio, left Atrial reservoir function; LSFP = left-sided filling pressure.

### Multivariable association with HFpEF and LSFP

In multivariable association analysis, LEDA was the echocardiographic variable of interest and remained independently associated with HFpEF after adjustment for age, sex, atrial fibrillation, hypertension, diabetes mellitus type 2, coronary artery disease, body mass index, and estimated glomerular filtration rate (Figure 3B). Each 1-point increase in LEDA was associated with approximately five-fold higher odds of HFpEF (OR: 5.35; 95% CI: 3.10-9.21; *P* < 0.001).

LEDA also remained independently associated with elevated invasive LSFP ≥15 mm Hg and with continuous invasive LSFP after adjustment for atrial fibrillation and clinical covariates. Detailed results are provided in Supplemental Table 3. The LEDA-by-atrial fibrillation interaction was not statistically significant; however, this analysis was exploratory and limited by subgroup size. Rhythm-stratified performance was broadly preserved in patients without and with any atrial fibrillation history (AUC: 0.84 and 0.85, respectively) (Supplemental Table 4).

### Diagnostic performance and validation

LEDA showed a stronger diagnostic performance for HFpEF than for H_2_FPEF and HFA-PEFF (Figure 2, Table 2). Compared with ASE 2025 criteria, LEDA also showed stronger discrimination, recognizing that ASE is a filling-pressure framework rather than a standalone HFpEF diagnostic score. The AUC for clinical HFpEF was 0.85 (95% CI: 0.79-0.91), compared with 0.74 for H_2_FPEF, 0.74 for HFA-PEFF, and 0.61 for ASE (*P* ≤ 0.010 for all). At LEDA ≥3, specificity was 89.9%, LR+ 7.43, LR− 0.28, and accuracy 83.6%. For invasive LSFP endpoints independent of LEDA components, AUCs were 0.90 for LSFP >12 mm Hg and 0.87 for LSFP ≥15 mm Hg, with specificity up to 95.5% and LR+ up to 16.3.

**Figure 2:**
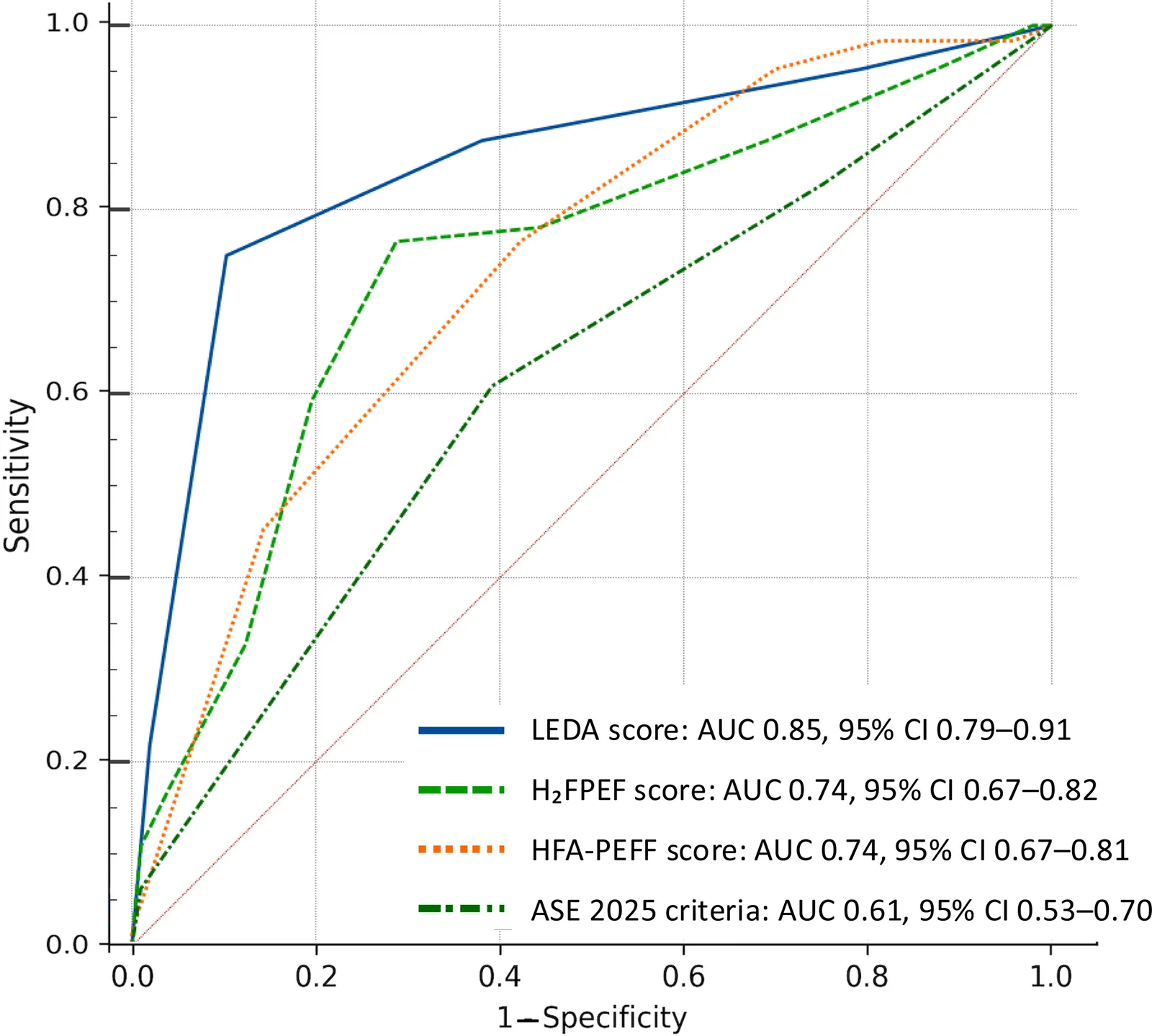
Receiver Operating Characteristic Analysis for Heart Failure With Preserved Ejection Fraction Diagnosis LEDA showed stronger discrimination in this selected cohort (AUC: 0.85; 95% CI: 0.79-0.91) than H_2_FPEF (0.74), HFA-PEFF (0.74), and ASE 2025 criteria analyzed as a filling-pressure framework (0.61). ASE = American Society of Echocardiography; AUC = area under the curve; HFA-PEFF = Heart Failure Association Pretest assessment, Echocardiography & natriuretic peptide, Functional testing, Final etiology diagnostic algorithm; H_2_FPEF = Heavy, Hypertensive, Atrial fibrillation, Pulmonary hypertension, Elder, Filling pressure score; LEDA = Left ventricular concentricity, E/e′, left ventricle-to-left atrium Diastasis volume ratio, left Atrial reservoir function.

**Table 2:** Diagnostic Performance of LEDA Compared With Established Clinical and Guideline-Based Scores

Outcome	Score	Threshold	AUC (95% CI)	Sensitivity (95% CI)	Specificity (95% CI)	LR+ (95% CI)	LR− (95% CI)	Accuracy (%)
Clinical HFpEF	LEDA	≥3	0.85 (0.79-0.91)	75.0% (63.9-83.6)	89.9% (82.4-94.4)	7.43 (4.06-13.56)	0.28 (0.19-0.42)	83.6
Clinical HFpEF	H_2_FPEF	≥6	0.74 (0.67-0.82)	59.7% (48.2-70.3)	79.8% (70.8-86.5)	2.96 (1.91-4.57)	0.50 (0.37-0.68)	71.3
Clinical HFpEF	HFA-PEFF	≥5	0.74 (0.67-0.81)	79.2% (68.4-86.9)	56.6% (46.7-65.9)	1.82 (1.41-2.35)	0.37 (0.23-0.60)	66.1
Clinical HFpEF	ASE 2025	Grade 2–3	0.61 (0.53-0.70)	60.9% (48.7-71.9)	60.8% (50.9-69.9)	1.56 (1.13-2.13)	0.64 (0.45-0.91)	60.9
LSFP >12 mm Hg	LEDA	≥3	0.90 (0.85-0.94)	73.2% (62.7-81.6)	95.5% (89.0-98.2)	16.28 (6.19-42.80)	0.28 (0.20-0.40)	84.8
LSFP >12 mm Hg	H_2_FPEF	≥6	0.77 (0.69-0.84)	58.5% (47.7-68.6)	83.1% (74.0-89.5)	3.47 (2.11-5.70)	0.50 (0.38-0.66)	71.3
LSFP >12 mm Hg	HFA-PEFF	≥5	0.71 (0.63-0.78)	75.6% (65.3-83.6)	57.3% (46.9-67.1)	1.77 (1.35-2.32)	0.43 (0.28-0.65)	66.1
LSFP >12 mm Hg	ASE 2025	Grade 2–3	0.61 (0.53-0.69)	59.5% (48.1-69.9)	62.1% (51.6-71.5)	1.57 (1.13-2.18)	0.65 (0.47-0.90)	60.9
LSFP ≥15 mm Hg	LEDA	≥3	0.87 (0.80-0.92)	81.7% (70.1-89.4)	86.5% (78.9-91.6)	6.04 (3.72-9.82)	0.21 (0.12-0.36)	84.8
LSFP ≥15 mm Hg	H_2_FPEF	≥6	0.74 (0.66-0.82)	61.7% (49.0-72.9)	76.6% (67.9-83.5)	2.63 (1.78-3.89)	0.50 (0.36-0.70)	71.3
LSFP ≥15 mm Hg	HFA-PEFF	≥5	0.70 (0.62-0.77)	78.3% (66.4-86.9)	52.3% (43.0-61.3)	1.64 (1.30-2.08)	0.41 (0.25-0.69)	61.4
LSFP ≥15 mm Hg	ASE 2025	Grades 2-3	0.60 (0.51-0.69)	59.3% (46.0-71.3)	57.9% (48.5-66.9)	1.41 (1.03-1.93)	0.70 (0.49-1.01)	58.4

Values are shown as estimates with 95% CIs where applicable. Area under the curve CIs were estimated using bootstrap resampling.

ASE = American Society of Echocardiography; AUC = area under the curve; H_2_FPEF = Heavy, Hypertensive, Atrial fibrillation, Pulmonary hypertension, Elder, Filling pressure score; HFA-PEFF = Heart Failure Association Pretest assessment, Echocardiography & natriuretic peptide, Functional testing, Final etiology diagnostic algorithm; HFpEF = heart failure with preserved ejection fraction; LEDA = left ventricular concentricity, E/e′, LV-to-LA diastasis volume ratio, and LA reservoir function; LR+ = positive likelihood ratio; LR− = negative likelihood ratio; LSFP = left-sided filling pressure.

In the 118-patient prospective validation cohort, LEDA retained strong performance for clinical HFpEF (AUC: 0.90), compared with H_2_FPEF (0.76), HFA-PEFF (0.76), and ASE 2025 criteria (0.65) (Supplemental Table 2). At LEDA ≥3, sensitivity was 82.2%, specificity 89.0%, LR+ 7.50, LR− 0.20, and accuracy 86.4%. LEDA AUCs for validation-cohort LSFP endpoints were 0.92 for LSFP >12 mm Hg and 0.93 for LSFP ≥15 mm Hg.

### Clinical utility and exploratory reclassification

Reclassification analyses were exploratory and are presented descriptively. Addition of LEDA to the clinical model was associated with improved integrated discrimination (integrated discrimination improvement: 0.14; 95% CI: 0.06-0.23), while overall net reclassification improvement was not emphasized because event and nonevent components were not the primary focus of this analysis. The decision curve analysis showed greater net benefit for LEDA than the clinical model across clinically relevant thresholds in this selected cohort (Figure 3A).

**Figure 3:**
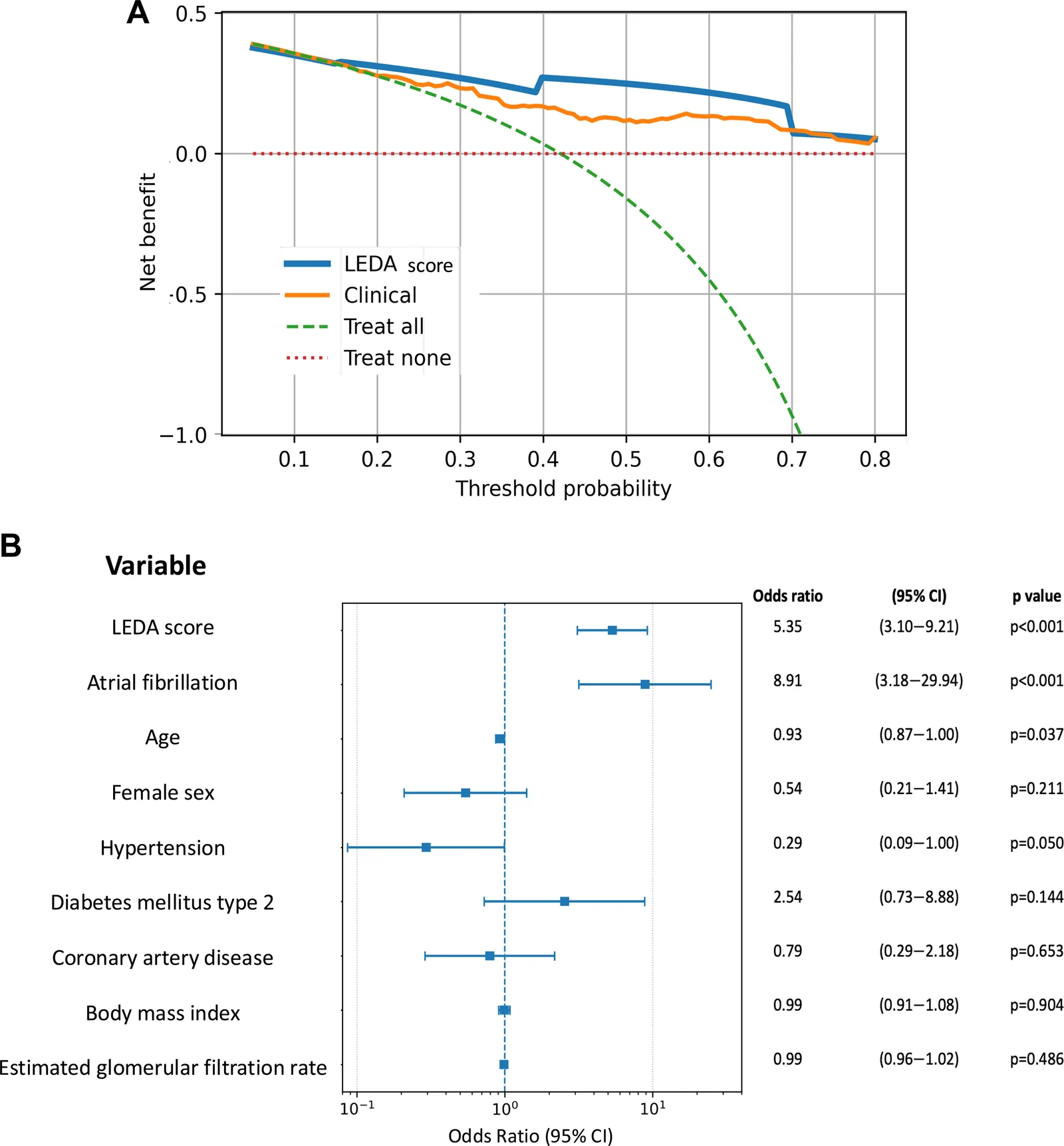
Clinical Utility and Multivariable Association Analysis (A) Decision curve analysis comparing LEDA with the clinical model. LEDA demonstrated greater net clinical benefit than the clinical model across clinically relevant threshold probabilities in this selected cohort. (B) Multivariable association analysis showing factors associated with heart failure with preserved ejection fraction after adjustment for clinical covariates. Odds ratios are shown with 95% confidence intervals. LEDA was included as the echocardiographic variable of interest; age, sex, atrial fibrillation, hypertension, diabetes mellitus type 2, coronary artery disease, body mass index, and estimated glomerular filtration rate were included as adjustment covariates. LEDA = Left ventricular concentricity, E/e′, left ventricle-to-left atrium Diastasis volume ratio, left Atrial reservoir function.

### LEDA distribution, component patterns, and reproducibility

LEDA scores were higher in patients with HFpEF than in those without HFpEF (median: 3.0 [2.8-3.0] vs 1.0 [1.0-2.0]; *P* < 0.001). Scores 3-4 occurred in 54/72 patients with HFpEF (75.0%) vs 10/99 patients without HFpEF (10.1%), whereas scores 0-1 occurred in 61/99 patients without HFpEF (61.6%) vs 8/72 patients with HFpEF (11.1%).

Among components, abnormal E/e′ was most common (70.2%), followed by LV concentricity (50.9%), abnormal LV-LA diastasis coupling (47.4%), and reduced LA reservoir function (26.3%). Each component was more frequent in HFpEF, with LV-LA diastasis coupling showing the largest separation (84.7% vs 20.2%). Component overlap was partial; reduced LA reservoir function was not strongly associated with LV concentricity or E/e′ alone, supporting nonredundancy. A reproducibility analysis of LV-LA diastasis volume ratio showed excellent intraobserver and good interobserver reproducibility (Supplemental Table 5).

Among patients classified as intermediate probability by H_2_FPEF (scores 2-5), HFA-PEFF (scores 2-4), or ASE 2025 LA pressure grade 1, LEDA reassigned 72.0%, 79.0%, and 75.5%, respectively, into low- or high-probability groups, with concordance with final HFpEF status of 85.1%, 83.7%, and 97.3%.

## Discussion

In this prospective, invasively validated cohort of selected patients with unexplained dyspnea and diagnostic uncertainty, LEDA showed stronger diagnostic performance for clinical HFpEF and invasive LSFP elevation than contemporary HFpEF diagnostic algorithms and an echocardiographic filling-pressure framework (Central Illustration). These findings should be interpreted within the context of a selected referral population enriched for HFpEF, comorbidity burden, and hemodynamic abnormality.

**Central Illustration:**
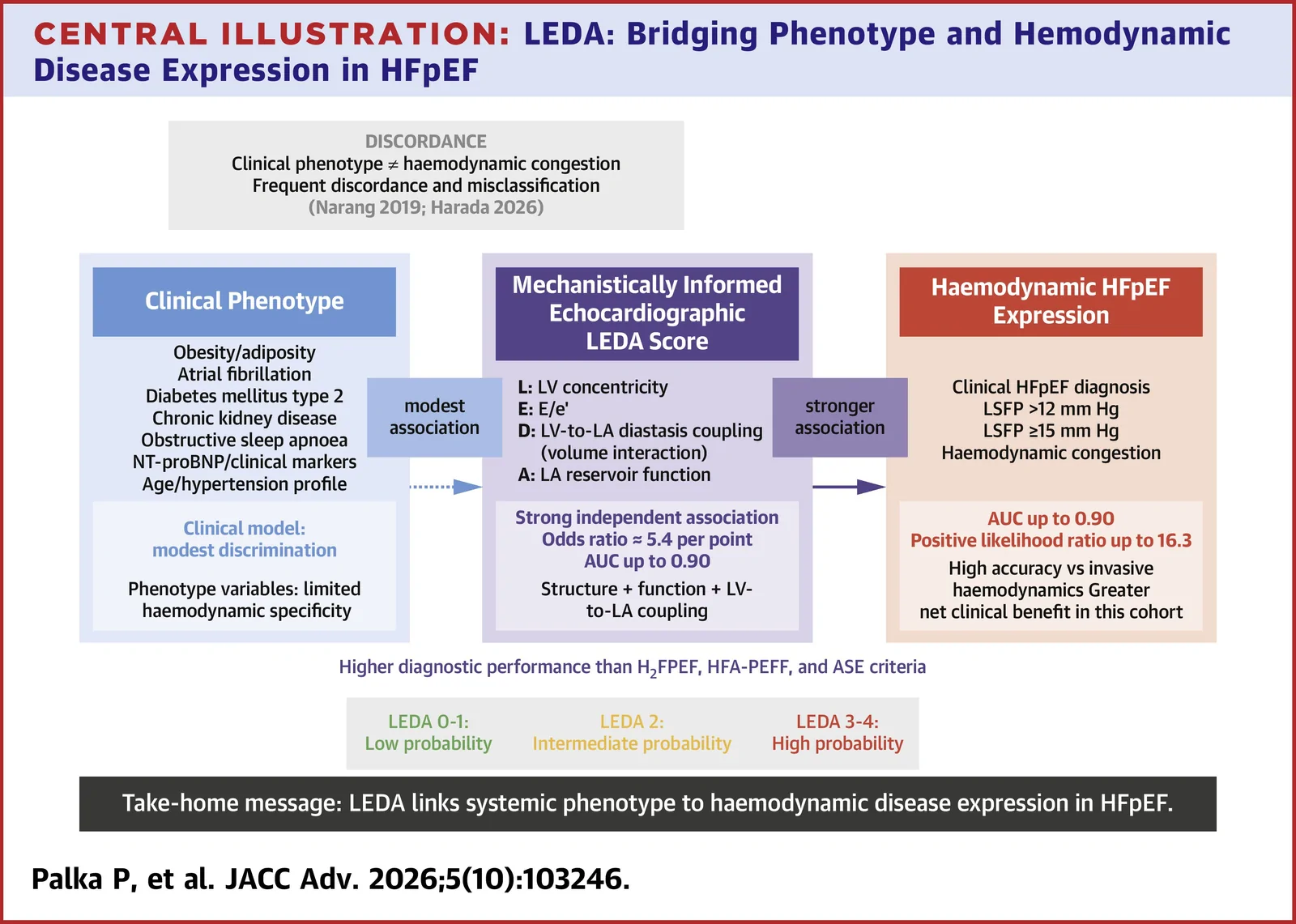
LEDA: Bridging Phenotype and Hemodynamic Disease Expression in HFpEF In this selected cohort, LEDA linked clinical phenotype to hemodynamic congestion by integrating LV concentricity, E/e′, LV-to-LA diastasis coupling, and LA reservoir function. Risk categories were LEDA 0-1 low, 2 intermediate, and 3-4 high probability. ASE = American Society of Echocardiography; AUC = area under the curve; HFA-PEFF = Heart Failure Association Pretest assessment, Echocardiography & natriuretic peptide, Functional testing, Final etiology diagnostic algorithm; HFpEF = heart failure with preserved ejection fraction; H_2_FPEF = Heavy, Hypertensive, Atrial fibrillation, Pulmonary hypertension, Elder, Filling pressure score; LA = left atrial; LEDA = left ventricular concentricity; LSFP = left-sided filling pressure; LV = left ventricular; NT-proBNP = N-terminal pro–B-type natriuretic peptide.

The present findings address an important limitation of current HFpEF diagnostic pathways: Noninvasive assessment and hemodynamic status are often discordant. Previous studies have shown a mismatch among clinical assessment, echocardiographic indexes, and directly measured filling pressures, even among experienced clinicians.^3^^,^^7^ H_2_FPEF and HFA-PEFF provide a structured diagnostic assessment but may leave patients in intermediate-probability categories, generate discordant classifications, and perform less consistently in selected phenotypes, including atrial fibrillation.^4^^,^^5^^,^^9^, ^10^, ^11^ The ASE diastolic algorithm should be interpreted differently from H_2_FPEF and HFA-PEFF because it is designed to estimate diastolic function and LA pressure, rather than to serve as a standalone HFpEF diagnostic score.^6^ Nevertheless, ASE criteria were included because estimation of the LSFP is central to HFpEF assessment, and recent invasive validation has highlighted limitations of resting diastolic grading when used in isolation.^8^

The diagnostic signal observed with LEDA is biologically plausible. HFpEF is increasingly recognized as a systemic, comorbidity-driven syndrome associated with obesity, atrial fibrillation, chronic kidney disease, inflammation, and adverse cardiometabolic remodeling.^32^, ^33^, ^34^, ^35^, ^36^, ^37^ Phenotype-based frameworks, phenomapping, and emerging artificial intelligence or machine-learning echocardiographic approaches have improved biological classification and may assist therapeutic targeting.^38^, ^39^, ^40^, ^41^, ^42^, ^43^ However, these approaches do not necessarily quantify filling pressure. LEDA was designed to complement, not replace, existing diagnostic tools by integrating variables that reflect several components of the filling-pressure pathway: LV geometry, Doppler-derived filling indexes, LV-LA diastasis coupling, and LA reservoir function.

Our previous hypertensive heart disease study supports this framework but addressed a different disease stage.^31^ The thresholds derived in that work reflected earlier stage B left-heart remodeling and were not intended as diagnostic cut-points for symptomatic HFpEF. In contrast, the present study used more stringent thresholds selected for stage C symptomatic HFpEF with elevated LSFP, suggesting that LEDA component thresholds may be disease-stage specific (Table 3).

**Table 3:** Relationship Between Previously Reported Hypertensive Heart Disease Thresholds and the Current LEDA Score Components

	Hypertensive Heart Disease Stage B	Clinical HFpEF Stage C	Abnormal Direction
LV end-diastolic volume index-to-mean LV wall thickness ratio	≤57	<50	Lower
E/e’ ratio	≥8	>9	Higher
LV diastasis volume-to-LA diastasis volume ratio	≤1.62	<1.25	Lower
LA reservoir function, fraction	≤0.58	<0.40	Lower

Thresholds are derived from the authors’ previous hypertensive heart disease study^41^ and are shown to support the biological continuity between early hypertensive remodeling and the LEDA framework used in the present HFpEF cohort.

E/e′ = ratio of early transmitral inflow velocity to early mitral annular velocity; HFpEF = heart failure with preserved ejection fraction; LA = left atrial; LEDA = left ventricular concentricity, E/e′, LV-to-LA diastasis volume ratio, and LA reservoir function; LV = left ventricular.

In the present cohort, LEDA demonstrated higher AUC values and stronger LRs than H_2_FPEF, HFA-PEFF, and ASE criteria for both clinical HFpEF and invasive LSFP endpoints. Reclassification analyses were exploratory and are interpreted descriptively; decision curve analysis indicated greater net clinical benefit across clinically relevant threshold probabilities in this cohort. In multivariable association analysis, LEDA remained independently associated with HFpEF after adjustment for atrial fibrillation, hypertension, type 2 diabetes mellitus, coronary artery disease, body mass index, renal function, age, and sex. The absence of a statistically significant LEDA-by-atrial fibrillation interaction should not be interpreted as definitive evidence that rhythm status has no effect; however, rhythm-stratified analyses suggested broadly preserved diagnostic performance, although larger external cohorts are required. Additional component-pattern analyses showed that the 4 LEDA domains were only partly overlapping, supporting their nonredundant contribution to the overall score. LEDA also reassigned a substantial proportion of patients classified as intermediate probability by H_2_FPEF, HFA-PEFF, or ASE criteria into low- or high-probability groups, but these reclassification findings should be considered exploratory.

Although LV and LA strain were assessed, strain parameters were not incorporated into LEDA. This decision was based on feasibility and conceptual considerations. LA strain is a sensitive marker of LA dysfunction, but a reliable measurement depends on image quality, tracking performance, vendor/software implementation, and rhythm stability.^44^ In this cohort, poor or severely limited image quality was present in 26% of patients, limiting feasibility in some cases. A volume-derived LA reservoir function measure was therefore used because it captures atrial reservoir performance but is simpler and less dependent on speckle-tracking analysis.^29^, ^30^, ^31^

The LV-LA diastasis volume ratio was the most discriminating individual LEDA component, but also the most novel and technically demanding. Unlike H_2_FPEF, which is simple to calculate from clinical variables, LEDA requires standardized echocardiographic acquisition, careful frame selection, and reader experience. This may limit immediate generalizability, particularly in patients with poor acoustic windows, atrial fibrillation, tachycardia, or suboptimal apical imaging. However, a reproducibility analysis from the present cohort showed excellent intraobserver and good interobserver reproducibility, supporting feasibility when performed using a standardized protocol.

Clinically, LEDA may provide a practical noninvasive estimate of filling-pressure burden in selected patients with unexplained dyspnea and diagnostic uncertainty, especially those with intermediate probability by current tools. LEDA should not be viewed as a replacement for an invasive hemodynamic assessment, particularly when clinical uncertainty remains high or exertional hemodynamics are required. Rather, it may help refine patient selection for further testing and provide a structured echocardiographic framework for suspected HFpEF. External validation is required before application to lower-prevalence outpatient echocardiography populations or patients with milder disease.

### Study limitations

First, although a derivation cohort was followed by prospective validation, both cohorts were derived from a single center, and thresholds were informed by prior work from the same investigative group; multicenter external validation is required. Second, the selected referral design enriched the cohort for HFpEF, comorbidity burden, and hemodynamic abnormality, so diagnostic performance and clinical utility may not directly translate to lower-prevalence outpatient populations. Third, incorporation bias is possible because the clinical HFpEF endpoint included elevated filling pressure, and LEDA was designed to reflect the filling-pressure burden. To address this, invasive LSFP endpoints were analyzed separately, and LSFP >12 mm Hg was examined as a complementary threshold. Fourth, LV-LA diastasis coupling is technically demanding and may be affected by image quality, atrial fibrillation, beat-to-beat variability, and local expertise despite good reproducibility in this cohort. Fifth, rhythm-stratified and interaction analyses were limited by subgroup size. Sixth, normal reference ranges for novel LEDA parameters are not established in large healthy populations. Although bootstrap resampling was used to assess the stability of estimates, the study was not designed as a prediction-model development study, and optimism-corrected performance estimates should be confirmed in external cohorts. Finally, no formal a priori sample-size calculation was performed for the full LEDA score, and confidence intervals for some secondary estimates were wide.

## Conclusions

In this prospective single-center cohort of selected patients with unexplained dyspnea undergoing invasive hemodynamic assessment for diagnostic uncertainty, LEDA showed stronger diagnostic performance than established HFpEF diagnostic algorithms and stronger discrimination of invasive LSFP than the ASE echocardiographic filling-pressure framework. External validation in broader, lower-prevalence outpatient populations is required to confirm generalizability, threshold reproducibility, and clinical utility.

Perspectives **COMPETENCY IN MEDICAL KNOWLEDGE:** Patients with suspected HFpEF often demonstrate discordance between clinical phenotype and hemodynamic disease expression. LEDA integrates LV structure, diastolic function, ventricular-atrial coupling, and LA reservoir function to provide a mechanistically informed echocardiographic assessment of elevated LSFP and HFpEF probability.

**TRANSLATIONAL OUTLOOK:** Prospective multicenter validation in broader outpatient and lower-prevalence populations is needed to determine whether a LEDA-guided assessment improves diagnostic pathways, reduces unnecessary invasive testing, and identifies patients with hemodynamically expressed HFpEF for targeted treatment strategies.

## Funding support and author disclosures

The authors have reported that they have no relationships relevant to the contents of this paper to disclose.
