# Supplementary material for: The LEDA Score: An Integrated Echocardiographic Score for the Diagnosis of HFpEF
Source: JACC Adv. 2026 Sep 18;5(10):103246. doi: 10.1016/j.jacadv.2026.103246 (PMC13627135; doi:10.1016/j.jacadv.2026.103246)
Supplement: Supplementary file 1 [file mmc1.docx]

**Supplementary Material**

**Article title**: *LEDA: An Integrated Echocardiographic Score for the Diagnosis of HFpEF*

**Corresponding Author**: Przemyslaw Palka

**Contents**:

1. Supplementary Figure 1, page 2
2. Supplementary Text, page 3
3. Supplementary Table 1, pages 4-7
4. Supplementary Table 2, page 8
5. Supplementary Table 3, pages 9,10
6. Supplementary Table 4, page 11
7. Supplementary Table 5, page 12

**Supplementary Text.**

**Relative Exclusion Criteria for Invasive Hemodynamic Assessment.**

- Active infection or sepsis (including respiratory infection or infective endocarditis)
- Prosthetic tricuspid valve
- Presence of right heart thrombus
- Severe coagulopathy or thrombocytopenia (INR >2.0 or platelet count <50,000/μL)
- Uncontrolled arrhythmias (e.g., ventricular tachycardia, atrial fibrillation, or atrial flutter with ventricular rate >100 beats/min)
- Severe uncontrolled hypertension
- Acute coronary syndrome
- Hemodynamic instability requiring inotropic support, mechanical circulatory support, or intensive intravenous therapy
- Acute respiratory failure requiring non-invasive or invasive ventilatory support
- Active acute kidney injury
- Severe anemia (hemoglobin <80 g/L)
- Primary severe or acute intracardiac valvular disease
- History of stroke within the preceding 6 months
- Acute heart failure associated with active cancer therapy (e.g., chemotherapy or immunotherapy)
- Patients receiving palliative care for advanced malignancy
- Pregnancy

**Note**

In selected complex patients (e.g., previous permanent pacemaker, suspected superior vena cava syndrome, or difficult venous access), direct left atrial pressure measurement using a retrograde approach was performed instead of right heart catheterization.

**Supplementary Table 1**. Echocardiographic comparison according to HFpEF status.

|  | **No HFpEF** | |  | **HFpEF** | |  |  |
| --- | --- | --- | --- | --- | --- | --- | --- |
| Parameter | Mean ± SD | 95% CI | | Mean ± SD | 95% CI | | p value |
| M-mode: |  |  | |  |  | |  |
| Interventricular septal wall thickness, cm | 0.95 ± 0.17 | 0.91–0.98 | | 1.05 ± 0.21 | 1.00–1.10 | | <0.001 |
| LV posterior wall thickness, cm | 0.90 ± 0.16 | 0.87–0.93 | | 1.01 ± 0.21 | 0.96–1.06 | | <0.001 |
| Mean wall thickness, cm | 0.93 ± 0.15 | 0.90–0.96 | | 1.03 ± 0.19 | 0.98–1.08 | | <0.001 |
| LV diameter, cm | 4.58 ± 0.59 | 4.46–4.70 | | 4.59 ± 0.75 | 4.41–4.76 | | 0.934 |
| Two-dimensional: |  |  | |  |  | |  |
| LV end-diastolic volume, mL | 90.65 ± 25.35 | 85.59–95.71 | | 87.83 ± 23.84 | 82.23–93.44 | | 0.463 |
| LV end-diastolic volume index, mL/m^2^ | 47.89 ± 10.93 | 45.71–50.07 | | 45.46 ± 10.98 | 42.88–48.04 | | 0.154 |
| LV diastasis volume, mL | 68.19 ± 20.11 | 64.18–72.20 | | 63.78 ± 23.54 | 58.25–69.31 | | 0.189 |
| LV diastasis volume index, mL/m^2^ | 36.10 ± 9.38 | 34.23–37.97 | | 32.96 ± 11.08 | 30.35–35.56 | | 0.047 |
| LV end-systolic volume, mL | 34.97 ± 11.64 | 32.65–37.29 | | 36.19 ± 13.75 | 32.96–39.43 | | 0.530 |
| LV stroke volume, mL | 55.68 ± 16.17 | 52.46–58.91 | | 51.60 ± 12.37 | 48.69–54.51 | | 0.075 |
| LV stroke volume index, mL/m^2^ | 29.47 ± 7.15 | 28.05–30.90 | | 26.82 ± 5.94 | 25.42–28.22 | | 0.011 |
| LV ejection fraction, % | 61.89 ± 5.38 | 60.81–62.96 | | 59.62 ± 6.00 | 58.21–61.04 | | 0.011 |
| LA maximum volume, mL | 66.60 ± 23.49 | 61.91–71.28 | | 86.37 ± 33.44 | 78.51–94.23 | | <0.001 |
| LA maximum volume index, mL/m^2^ | 35.48 ± 12.20 | 33.05–37.92 | | 44.94 ± 16.92 | 40.97–48.92 | | <0.001 |
| LA diastasis volume, mL | 45.95 ± 15.46 | 42.87–49.04 | | 70.26 ± 26.31 | 64.08–76.45 | | <0.001 |
| LA diastasis volume index, mL/m^2^ | 24.51 ± 8.15 | 22.88–26.13 | | 36.54 ± 13.49 | 33.37–39.71 | | <0.001 |
| LA minimum volume, mL | 28.89 ± 14.60 | 25.97–31.80 | | 51.57 ± 29.29 | 44.69–58.45 | | <0.001 |
| LA minimum volume index, mL/m^2^ | 15.46 ± 7.95 | 13.88–17.05 | | 26.58 ± 14.29 | 23.23–29.94 | | <0.001 |
| LV concentricity / hypertrophy: |  |  | |  |  | |  |
| Relative wall thickness | 0.41 ± 0.09 | 0.39–0.43 | | 0.46 ± 0.13 | 0.43–0.49 | | 0.002 |
| LV mass, g | 144.87 ± 45.34 | 135.83–153.91 | | 168.60 ± 55.61 | 155.53–181.66 | | 0.002 |
| LV mass index, g/m^2^ | 76.51 ± 20.25 | 72.47–80.54 | | 86.54 ± 23.91 | 80.92–92.16 | | 0.003 |
| LV mass-to-end-diastolic volume ratio | 1.66 ± 0.53 | 1.56–1.77 | | 1.94 ± 0.53 | 1.82–2.07 | | <0.001 |
| Doppler and Strain: |  |  | |  |  | |  |
| LV global longitudinal strain, % * | 18.52 ± 3.02 | 17.86–19.18 | | 16.69 ± 3.88 | 15.56–17.81 | | 0.003 |
| LA reservoir strain, % * | 26.59 ± 10.31 | 24.23–28.94 | | 17.21 ± 10.20 | 14.40–20.02 | | <0.001 |
| E-wave, cm/s | 72 ± 19 | 68–76 | | 89 ± 33 | 82–97 | | <0.001 |
| E-wave deceleration time, ms | 219 ± 49 | 210–229 | | 204 ± 65 | 189–219 | | 0.100 |
| A-wave, cm/s (sinus rhythm) | 79 ± 25 | 74–84 | | 81 ± 31 | 73–89 | | 0.681 |
| E/A ratio (sinus rhythm) | 0.99 ± 0.51 | 0.89–1.10 | | 1.17 ± 0.57 | 1.01–1.32 | | 0.056 |
| Isovolumic relaxation time, ms * | 95 ± 21 | 90–101 | | 84 ± 31 | 75–93 | | 0.043 |
| Septal e’, cm/s | 6.08 ± 1.94 | 5.69–6.47 | | 5.69 ± 1.67 | 5.30–6.09 | | 0.175 |
| Lateral e’, cm/s | 7.82 ± 2.39 | 7.34–8.30 | | 7.95 ± 2.56 | 7.34–8.56 | | 0.750 |
| e’ (average), cm/s | 6.90 ± 2.06 | 6.49–7.32 | | 6.76 ± 1.93 | 6.30–7.22 | | 0.650 |
| E/e’ septal, ratio | 12.85 ± 5.25 | 11.80–13.90 | | 17.64 ± 11.75 | 14.88–20.40 | | <0.001 |
| E/e’ lateral, ratio | 10.01 ± 3.89 | 9.23–10.79 | | 13.31 ± 10.85 | 10.73–15.90 | | 0.006 |
| Tricuspid regurgitation velocity, m/s * | 2.47 ± 0.41 | 2.37–2.56 | | 2.66 ± 0.48 | 2.54–2.78 | | 0.009 |
| Estimated right ventricular systolic pressure, mmHg * | 32.73 ± 9.48 | 30.67–34.80 | | 37.56 ± 10.84 | 34.83–40.29 | | 0.005 |
| A-wave duration − atrial reversal duration, ms (sinus rhythm) * | 7.25 ± 30.06 | 0.38–14.12 | | -9.75 ± 38.02 | -22.61–3.11 | | 0.012 |
| S-wave / D-wave, ratio * | 1.42 ± 0.38 | 1.32–1.52 | | 1.11 ± 0.55 | 0.94–1.29 | | 0.002 |
| LEDA score components: |  |  | |  |  | |  |
| LV end-diastolic volume index-to-mean wall thickness, ratio | 52.79 ± 13.55 | 50.09–55.49 | | 45.44 ± 12.69 | 42.46–48.43 | | <0.001 |
| E/e' (average), ratio | 11.42 ± 4.34 | 10.56–12.29 | | 15.49 ± 10.97 | 12.91–18.06 | | 0.001 |
| LV diastasis volume-to-LA diastasis volume, ratio | 1.59 ± 0.63 | 1.47–1.72 | | 0.96 ± 0.34 | 0.88–1.05 | | <0.001 |
| LA reservoir function, fraction | 0.57 ± 0.15 | 0.54–0.60 | | 0.42 ± 0.20 | 0.37–0.46 | | <0.001 |

Continuous variables are presented as mean ± standard deviation with corresponding 95% confidence intervals. Between-group comparisons were performed using Student’s t-test or Mann–Whitney U test, as appropriate. *Indicates measurements with incomplete case numbers.

Abbreviations: CI = confidence interval; SD = standard deviation; HFpEF = heart failure with preserved ejection fraction; LA = left atrial; LV = left ventricular.

**Supplementary Table 2. Diagnostic performance of LEDA compared with H₂FPEF, HFA-PEFF, and ASE 2025 criteria in the prospective validation cohort.**

| **Validation cohort, n=118** | **LEDA** | **H₂FPEF** | **HFA-PEFF** | **ASE 2025** |
| --- | --- | --- | --- | --- |
| AUC for clinical HFpEF* | 0.90 | 0.76 | 0.76 | 0.65 |
| Sensitivity | 82.2% | 64.4% | 82.2% | 64.9% |
| Specificity | 89.0% | 79.5% | 60.3% | 62.0% |
| LR+ | 7.50 | 3.14 | 2.07 | 1.71 |
| LR- | 0.20 | 0.45 | 0.29 | 0.57 |
| Accuracy | 86.4% | 73.7% | 68.6% | 63.0% |

*, For haemodynamic endpoints in the validation cohort, LEDA AUC was 0.92 for LSFP >12 mmHg and 0.93 for LSFP ≥15 mmHg.

LR+, positive likelihood ratio; LR-, negative likelihood ratio.

**Supplementary Table 3**. Association of LEDA with HFpEF and invasive left-sided filling pressure after adjustment for atrial fibrillation status and clinical covariates.

| **Outcome** | **Atrial fibrillation definition** | **Model type** | **LEDA effect per 1-point increase** | **95% Confidence interval** | **LEDA p value** | **LEDA × atrial fibrillation interaction p value** |
| --- | --- | --- | --- | --- | --- | --- |
| HFpEF diagnosis | Any AF | Logistic regression | OR 5.35 | 3.10–9.21 | <0.001 | 0.735 |
| HFpEF diagnosis | Persistent/current AF | Logistic regression | **OR 4.68** | **2.88–7.59** | <0.001 | **0.254** |
| LSFP ≥15 mmHg | Any AF | Logistic regression | OR 7.81 | 4.09–14.91 | <0.001 | 0.090 |
| LSFP ≥15 mmHg | Persistent/current AF | Logistic regression | **OR 7.08** | **3.90–12.86** | <0.001 | **0.467** |
| Continuous LSFP | Any AF | Linear regression | β +2.67 mmHg | 2.07–3.27 | <0.001 | 0.407 |
| Continuous LSFP | Persistent/current AF | Linear regression | **β +2.90 mmHg** | **2.27–3.52** | <0.001 | **0.557** |

Values are derived from multivariable models adjusted for atrial fibrillation status and clinical covariates, including age, female sex, hypertension, diabetes mellitus type 2, coronary artery disease, body mass index, and estimated glomerular filtration rate. Logistic regression was used for HFpEF diagnosis and elevated LSFP ≥15 mmHg. Linear regression was used for continuous invasive LSFP. For logistic regression models, the LEDA effect estimate is shown as the odds ratio per 1-point increase in LEDA score. For continuous LSFP, β represents the change in invasive LSFP, in mmHg, per 1-point increase in LEDA score. “Any AF” included any history of atrial fibrillation, including paroxysmal, persistent, permanent, or chronic atrial fibrillation. “Persistent/current AF” referred to atrial fibrillation present at the time of hemodynamic and echocardiographic assessment. Interaction p values refer to the LEDA × atrial fibrillation interaction term.

**AF = atrial fibrillation; CI = confidence interval; HFpEF = heart failure with preserved ejection fraction; LSFP = left-sided filling pressure; OR = odds ratio.**

**Supplementary Table 4**. Rhythm-stratified diagnostic performance of LEDA for clinical HFpEF.

| **Rhythm subgroup** | **n** | **HFpEF, n** | **AUC** | **Sensitivity** | **Specificity** | **LR+** | **LR−** | **Accuracy** |
| --- | --- | --- | --- | --- | --- | --- | --- | --- |
| No atrial fibrillation history | 107 | 28 | 0.84 | 75.0% | 91.1% | 8.46 | 0.27 | 86.9% |
| Any atrial fibrillation history | 64 | 44 | 0.85 | 75.0% | 85.0% | 5.00 | 0.29 | 78.1% |

HFpEF = heart failure with preserved ejection fraction; AUC = area under the curve; LR+ = positive likelihood ratio; LR− = negative likelihood ratio.

**Supplementary Table 5**. Reproducibility of LV–LA diastasis volume ratio in the present cohort.

| **Parameter** | **Intra-observer** | **Inter-observer** |
| --- | --- | --- |
| Patients, n | 27 | 27 |
| Intraclass correlation coefficient | 0.97 | 0.91 |
| Mean bias | +0.01 | −0.03 |
| 95% limits of agreement | −0.26 to +0.29 | −0.47 to +0.42 |
| Coefficient of variation | 9.9% | 16.3% |
| Agreement for ratio <1.25 | 92.6% | 85.2% |
| Cohen κ for ratio <1.25 | 0.85 | 0.70 |

Reproducibility analysis of the LV–LA diastasis volume ratio in 27 patients from the present cohort demonstrated excellent intra-observer and good inter-observer reproducibility. The intra-observer intraclass correlation coefficient was 0.97, with mean bias +0.01 and 95% limits of agreement −0.26 to +0.29. The inter-observer intraclass correlation coefficient was 0.91, with mean bias −0.03 and 95% limits of agreement −0.47 to +0.42. Agreement for classification using the predefined abnormal threshold of <1.25 was 92.6% intra-observer and 85.2% inter-observer, with Cohen κ values of 0.85 and 0.70, respectively.
